# Business Closures, Stay-at-Home Restrictions, and COVID-19 Testing Outcomes in New York City

**DOI:** 10.5888/pcd17.200264

**Published:** 2020-09-17

**Authors:** George J. Borjas

**Affiliations:** 1 Harvard Kennedy School, Cambridge, Massachusetts

## Abstract

**Introduction:**

In response to the coronavirus disease 2019 (COVID-19) pandemic, New York City closed all nonessential businesses and restricted the out-of-home activities of residents as of March 22, 2020. This order affected different neighborhoods differently, as stores and workplaces are not randomly distributed across the city, and different populations may have responded differently to the out-of-home restrictions. This study examines how the business closures and activity restrictions affected COVID-19 testing results. An evaluation of whether such actions slowed the spread of the pandemic is a crucial step in designing effective public health policies.

**Methods:**

Daily data on the fraction of COVID-19 tests yielding a positive result at the zip code level were analyzed in relation to the number of visits to local businesses (based on smartphone location) and the number of smartphones that stayed fixed at their home location. The regression model also included vectors of fixed effects for the day of the week, the calendar date, and the zip code of residence.

**Results:**

A large number of visits to local businesses increased the positivity rate of COVID-19 tests, while a large number of smartphones that stayed at home decreased it. A doubling in the relative number of visits increases the positivity rate by about 12.4 percentage points (95% CI, 5.3 to 19.6). A doubling in the relative number of stay-at-home devices lowered it by 2.0 percentage points (95% CI, −2.9 to −1.2). The business closures and out-of-home activity restrictions decreased the positivity rate, accounting for approximately 25% of the decline observed in April and May 2020.

**Conclusion:**

Policy measures decreased the likelihood of positive results in COVID-19 tests. These specific policy tools may be successfully used when comparable health crises arise in the future.

**Summary**

**What is already known on this topic?**

Little is known about whether the business closures and restrictions on out-of-home activities mandated during the coronavirus disease 2019 (COVID-19) pandemic by many government units in the United States and abroad helped contain the spread of the virus.

**What is added by this report?**

This article examines daily testing data for New York City to determine if the economic and behavioral restrictions imposed by government policies limited the spread of COVID-19 in a dense urban setting.

**What are the implications for public health practice?**

These data suggest that the policy measures decreased the likelihood of positive results in COVID-19 tests. The study identifies specific policy tools that may be successfully used when comparable health crises arise in the future.

## Introduction

The New York metropolitan area quickly became the epicenter of the coronavirus disease 2019 (COVID-19) pandemic in the United States. The first test in New York City for severe acute respiratory syndrome coronavirus 2 (SARS-CoV-2), the virus that causes COVID-19, was administered on January 29, 2020, with the first positive result not confirmed until February 23, 2020 (1). By the end of March 2020, New York City had 67,789 people infected with the virus, and 2,193 people had died from the disease; by July 15, some 260,176 people had been infected and the total number of confirmed deaths was 18,756 (1).

These citywide statistics mask a lot of variation in testing outcomes across geographic areas in the city. Some New York City neighborhoods (as demarcated by zip code) were heavily affected while others were relatively unscathed. There is evidence that the initial testing resources were more readily available to people residing in wealthier neighborhoods and that people in those neighborhoods were less likely to test positive (2–5). In contrast, people residing in racial/ethnic minority neighborhoods, particularly neighborhoods with a large African American or Hispanic population, tended to test positive at much higher rates (2).

Effective on March 22, 2020, the state government issued a “New York State on PAUSE” executive order that closed all nonessential businesses, prohibited nonessential gatherings of individuals outside their homes, and limited outdoor recreational activities (6). The business closures affected different neighborhoods differently, as the location of stores and workplaces is not randomly distributed across New York City. Moreover, although government officials did not proclaim a stay-at-home order, the prohibition on nonessential gatherings effectively compelled people to spend a large fraction of their time at home. Different demographic or socioeconomic groups may differ in their propensities or opportunities to adhere to the curbs on out-of-home activities. These differences in the impact of the business closures or out-of-home activity restrictions may have created further geographic disparities in testing outcomes.

This study merges daily data on testing outcomes at the zip code level in New York City with information on the number of visitors to local points of interest (such as stores, restaurants, parks, hospitals, or museums) and the number of people who limited their out-of-home activities. Previous research on the spread of pandemic diseases, including COVID-19 and the 2009 H1N1 influenza, emphasizes the key role played by spatial diffusion (7–11). An understanding of the determinants of spatial transmission at the various stages of a pandemic is critical for the design of public health policies that seek to halt the spread of the pandemic or reduce the possibility of new outbreaks after the initial wave.

This study examines the geographic dispersion observed in the positivity rate across New York City neighborhoods to determine if the economic and behavioral restrictions imposed by the executive order limited the spread of COVID-19 in a dense urban setting. Such an evaluation can help identify which types of interventions are most effective in reducing the mortality and disease caused by pandemics. The evaluation can also inform the tradeoff between improved public health outcomes and the cost of limitations on social and economic activity.

## Methods

I conducted a statistical analysis of COVID-19 testing data compiled by the New York City Department of Health and Mental Hygiene (DOH) that provides information on test results at the zip code level (12). The data are available beginning on April 1, 2020, and give a daily cumulative count of the number of tests and positive results for residents in each of 177 zip codes in the city. The sample period used in this study ended on May 31, 2020 (just before the disruption in the social distancing protocols during the protests of late May and early June 2020 might affect testing outcomes).

I merged the testing data with smartphone location information compiled by SafeGraph, a private company that partners with mobile applications to collect location data from 35 million mobile devices. The inclusion of a mobile device in the SafeGraph sample is not random. It only includes those users who gave applications opt-in consent to collect anonymous location data (13). The location data report the daily number of visitors to specific places in each zip code and the mobility patterns of residents in the neighborhood. The study uses a regression model that exploits the variation in testing outcomes over time and across zip codes to establish if the business closures and out-of-home activity restrictions affected how COVID-19 spread through the city.

### Study measures

#### Positivity rate

Citywide data on the daily number of tests and number of positive tests are available from the New York City DOH. Beginning on April 1, 2020, DOH also began to release daily information on the cumulative number of tests administered and the cumulative number of positive results for people residing in each of 177 zip codes. The daily report often allocates a small number of tests to a nonidentifiable geographic area (on average, the zip code is missing for 1.2% of daily tests and 2.1% of positive results). The statistical analysis excludes the test results that were not allocated to a particular zip code.

The information reported by DOH is not complete. The agency did not release the cumulative counts on 1 day in the sample period, and the released data were not usable on 2 other days (eg, the cumulative counts reported for a given day were identical to or smaller than the cumulative counts reported the day before). I corrected these inconsistencies, which affected 4.9% of the observations in the sample period, by linearly interpolating the cumulative counts from the day before and the day after the missing data. Test information at the zip code level is not available for the critical month of March (the month when the virus began to spread rapidly and the executive order was issued).

The cumulative number of tests and positive results was converted into a daily number by differencing the day-to-day cumulative totals. The positivity rate on any given day is defined as the percentage of tests administered that day that yielded a positive result for COVID-19. This statistic is available daily from April 2, 2020, through May 31, 2020, for each zip code in the city.

#### Business activity index

I used the Weekly Patterns places data from SafeGraph to construct an index of the number of people who visited any point of interest in a zip code on any given day (14). The data available from SafeGraph report the number of smartphone devices that visited every point of interest in the zip code on any given day.

A business activity index was constructed by first adding up all visits on a given day across all points of interest in a zip code. This sum was then converted into a rate per 1,000 people in the zip code, where the zip code’s population is an intercensal estimate produced by the DOH and the Department of City Planning (12). To ease the interpretation of the results, the business activity index is normalized to equal 100 for the entire city in the prepandemic period of February 4 through 6. This normalization allows the value of the index for any zip code at any point in time to be interpreted as percentage deviations from the prepandemic citywide average.

The testing for COVID-19 in the first months of the pandemic was targeted at people who had developed specific symptoms. The median number of days from exposure to the onset of symptoms is estimated to be 5.1 days, with 72.5% of cases observed between 2.2 and 6.7 days (15). The regression analysis would then relate testing outcomes on any given day *t* to the average value of the business activity index in the zip code 3 to 7 days prior (eg, the testing outcomes on May 11 are related to the average value of the index between May 4 and May 8).

#### Stay-at-home index

I used SafeGraph’s Social Distancing Metrics data to construct an index that approximates the fraction of people in a neighborhood that stayed at home during the pandemic (16). SafeGraph assigns each smartphone device a “home location,” the most common nighttime location of that device over a prior 6-week period (with a precision of 100 m^2^). For each census block group, the SafeGraph data then reports the number of devices that did not leave their home location on any given day.

The data were aggregated to the zip code level using a crosswalk file produced by the US Department of Housing and Urban Development that allocates census tracts to zip codes (17). For each day–zip code combination, I defined the stay-at-home index as the number of devices that did not leave their home location per 1,000 people in the zip code. The stay-at-home index was also normalized to equal 100 for the entire city during February 4 through 6. The regression analysis would then relate the testing outcomes on any given day to the average value of the stay-at-home index 3 to 7 days prior.

### Statistical analysis

The analysis used a linear regression model to determine the association between the positivity rate for residents in a zip code on any given day and the lagged values of the business activity and stay-at-home indices. The data consisted of 1 observation per zip code per day from April 2 through May 31. The regression has 10,554 observations (177 zip codes each observed 60 days, minus the day–zip code combinations where no tests were administered).

The regression model also included vectors of fixed effects to net out other factors that affect the positivity rate. These additional regressors included a vector of day-of-week fixed effects (eg, Monday, Tuesday). The frequency of testing is typically lower on weekends, and the reported outcome of those tests may be delayed until the beginning of the work week. The regression included a vector of fixed effects giving the actual calendar date in which the test was given (eg, April 13 or May 5). These calendar date fixed effects help net out the citywide trend in the positivity rate. Finally, the regression included a vector of zip code fixed effects. These fixed effects net out factors that permanently affected the positivity rate in a particular neighborhood throughout the April–May period.

The inclusion of the zip code fixed effects controls for geographic differences in socioeconomic characteristics that are specific to the zip code and that did not change over the sample period. Put differently, although the regression does not include any neighborhood-specific socioeconomic status variables (such as ethnicity, race, sex, or income that could be calculated from the annual American Community Survey data), the impact of these characteristics is effectively subsumed by the zip code fixed effects.

## Results

The citywide trend in the test positivity rate in New York City reached a maximum of 71% on March 28 and declined steadily in the next 2 months (Figure). By May 31, only 4% of tests had a positive outcome. The business activity and stay-at-home indexes averaged across zip codes were near their prepandemic value of 100 until about the middle of March, just before the executive order went into effect (Figure). At that time, the business activity index rapidly declined and bottomed out on April 16 when it reached a low of 22.9. In contrast, the stay-at-home index rapidly increased, reaching a peak of 206.6 on April 8.

**Figure:**
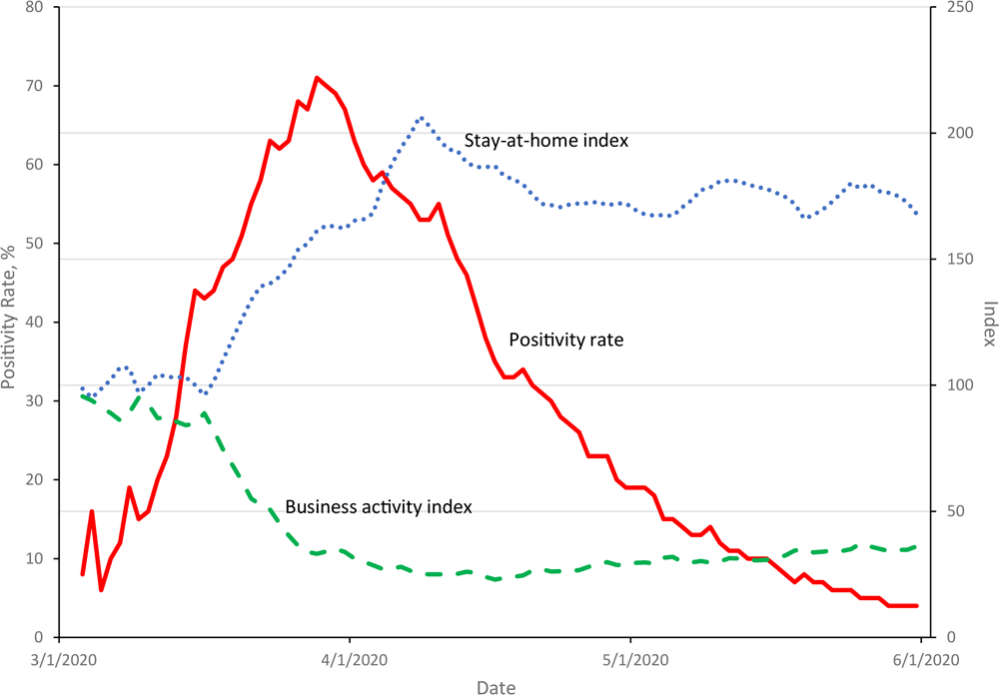
Citywide trends in the positivity rate for severe acute respiratory syndrome coronavirus 2 (SARS-CoV-2) and business activity and stay-at-home indices, New York City, March 3–May 31, 2020. The positivity rate gives the percentage of daily tests that had a positive result; the business activity index gives the number of visitors to points of interest (such places as stores, restaurants, parks, hospitals, or museums) in a zip code; and the stay-at-home index counts the number of smartphone devices that did not leave their home location. Both indices are averaged across zip codes (weighted by population), are lagged 3 to 7 days before the day of the test, and are normalized to equal 100 in the prepandemic period of February 4 through 6.

These citywide trends mask the large variance in testing outcomes across neighborhoods. Table 1 shows some of the variation by reporting the positivity rate at 3 different points in time for the most populous zip code in each of the 5 boroughs. To ensure comparability, all time periods refer to a Tuesday–Thursday time frame. The Manhattan neighborhood (Manhattan Valley/Morningside Heights/Upper West Side) had a relatively low positivity rate of 58.9% in early April. This contrasts with the 85.1% positivity rate in the Corona/North Corona neighborhoods of Queens.

**Table 1:** Trends in the Positivity Rate for the Most Populous Zip Code in Each Borough^a^, New York City, April–May 2020

Location	Positivity rate, %^b^		
	April 7–9	May 5–7	May 26–28
**Most populous zip code in**			
Manhattan	58.9	5.9	3.3
The Bronx	65.9	17.4	5.7
Queens	85.1	23.3	7.1
Brooklyn	63.9	9.1	2.7
Staten Island	46.8	13.9	5.9
**Citywide**	53.4	14.0	4.7

a Manhattan, zip code 10025 (Manhattan Valley/Morningside Heights/Upper West Side); The Bronx, 10467 (Allerton/Norwood/ Pelham Parkway/Williamsbridge); Queens, 11368 (Corona/North Corona); Brooklyn, 11211 (East Williamsburg/Williamsburg [North]/Williamsburg [South]); and Staten Island, 10314 (Bloomfield/Freshkills Park).

b The positivity rate gives the percentage of tests administered in a particular geographic area on a given day that yielded a positive result.

Table 1 also shows the geographic variation in the speed at which the positivity rate fell. The positivity rate in the most populous Bronx neighborhood (Allerton/Norwood/ Pelham Parkway/Williamsbridge) declined from 65.9 to 17.4 between early April and early May. In contrast, the rate in the Williamsburg neighborhood of Brooklyn started off at roughly the same level in April (63.9) but had dropped to 9.1 by early May.

Table 2 documents the large geographic differences in the business activity and stay-at-home indices, both at a point in time and in their rate of change as the pandemic took hold. The trend in the business activity index shows that the number of visitors to a point of interest in Manhattan fell by about 46 points (from 66 to 20) between February (before any mobility restrictions) and early May. The decline was less steep in Queens, where the index fell by 34 points (from 62 to 28). Similarly, the stay-at-home index rose faster in the Queens neighborhood than in the Bronx one. In Queens, the stay-at-home index increased from 89 to 163 between February and early May, while it rose from 100 to 144 in the Bronx.

**Table 2:** Business Activity and Stay-At-Home Indices for the Most Populous Zip Code in Each Borough^a^, New York City, February–May 2020

Index	February 4–6	April 7–9	May 5–7	May 26–28
**Business activity index^b^**				
Manhattan	66	18	20	24
The Bronx	74	27	33	42
Queens	62	23	28	35
Brooklyn	42	12	15	19
Staten Island	129	36	43	52
Citywide	100	26	32	39
**Stay-at-home index^c^**				
Manhattan	55	90	84	75
The Bronx	100	148	144	131
Queens	89	181	163	148
Brooklyn	37	54	53	47
Staten Island	100	245	232	204
Citywide	100	189	183	165

a Manhattan, zip code 10025 (Manhattan Valley/Morningside Heights/Upper West Side); The Bronx, 10467 (Allerton/Norwood/ Pelham Parkway/Williamsbridge); Queens, 11368 (Corona/North Corona); Brooklyn, 11211 (East Williamsburg/Williamsburg [North]/Williamsburg [South]); Staten Island, 10314 (Bloomfield/Freshkills Park).

b Average number of visits to a point of interest (such places as stores, restaurants, parks, hospitals, or museums) per 1,000 people in the zip code, normalized to equal 100 for the entire city in the prepandemic period of February 4–6.

c Average number of smartphone devices that did not leave the home location per 1,000 people in the zip code, normalized to equal 100 for the entire city in the prepandemic period of February 4–6.

The correlation coefficient between the business activity and stay-at-home indices is 0.39. This correlation is modest and suggests that multicollinearity between the 2 indices does not play a role in the estimation of the regression model. In contrast, the correlation between the lagged and current values of the indices is high: 0.94 for the business activity index and 0.92 for the stay-at-home index.

Both the business activity and stay-at-home indices have statistically significant effects on the positivity rate (Table 3). A 100-unit increase in the business activity index (implying a doubling in the relative number of visitors from the citywide prepandemic average) increased the positivity rate by 12.4 percentage points (*P* = .001; 95% CI, 5.3–19.6). A 100-unit increase in the stay-at-home index (implying a doubling in the relative number of devices that did not leave the home location) decreased the positivity rate by 2.0 percentage points (*P* < .001; 95% CI, −2.9 to −1.2). The regression model explains 82% of the variation in the positivity rate across neighborhoods and over time (Table 3).

**Table 3:** Determinants of the Positivity Rate of Tests for Severe Acute Respiratory Syndrome Coronavirus 2 (SARS-CoV-2) (N = 10,554), New York City, April–May 2020

Regressor^a^	Mean (SD)	β^b^ (95% CI)	*P* Value
Lagged business activity index	33.1 (22.6)	0.124 (0.053 to 0.196)	.001
Lagged stay-at-home index	192.1 (93.2)	−0.020 (−0.029 to −0.012)	<.001
*R* ^2^	NA	0.824	NA

Abbreviation: NA, not applicable; SD, standard deviation.

a The business activity index gives the average number of visits to a point of interest (such places as stores, restaurants, parks, hospitals, or museums) per 1,000 people in the zip code. The stay-at-home index gives the average number of smartphone devices that did not leave the home location per 1,000 people. Both indices are normalized to equal 100 for the entire city in the prepandemic period of February 4–6. The regression uses the average lagged value of the indices 3 to 7 days before the administration of the test.

b Regression coefficient from linear regression that also includes day-of-week, calendar date, and zip code fixed effects. The dependent variable gives the daily percentage of tests administered to residents of a zip code that gave a positive result (mean [SD], 26.4 [23.1]). The standard error of β is clustered at the zip code level. The regression excludes zip code–day combinations where no tests were administered.

The positivity rate in the city decreased from about 54% in early April to 14% in early May (Table 1). The business activity index decreased by about 70 points from the prepandemic baseline to early May, producing a 9-percentage point drop in the positivity rate. The stay-at-home index increased by about 80 points, producing a 2-percentage point drop in the positivity rate. The total direct impact of the 2 indices, therefore, accounted for approximately 25% of the observed 40-percentage point decline in the positivity rate.

## Discussion

The regression analysis indicates that the nonessential business closures and out-of-home activity restrictions adopted in New York City decreased the positivity rate of COVID-19 tests. The quantitative size of the impact, however, was relatively small.

The regression model explains 82% of the variation in the positivity rate across neighborhoods and over time, but the 2 indices accounted for only 25% of the drop in the average positivity rate for the city. The positivity rate varied considerably across New York City neighborhoods and declined noticeably during the sample period. The zip code fixed effects explain a large part of this cross-section variation, and several studies suggest that it is partly attributable to neighborhood differences in such variables as household income and racial composition (2–5). At the same time, the calendar date fixed effects net out the steep citywide decline. The 2 sets of fixed effects help produce the large explanatory power of the regression.

The inclusion of the fixed effects in the regression model implies that the impact of the business activity and stay-at-home indices is identified by correlating the indices with the positivity rate (net of the citywide trend) within a specific zip code. Put differently, the regression coefficients only capture the impact of a change in the local index on the net positivity rate of the typical zip code. The indices could account for more of the citywide trend if there were substantial “spillovers” across neighborhoods. A change in the indices in one zip code would then affect the positivity rate in other zip codes, and the spatial autocorrelation might generate part of the citywide decline. A more detailed examination of the SafeGraph data, taking into account the geographic origin of visitors or the stay-at-home decisions of residents in nearby neighborhoods, could potentially be used to directly estimate the spatial autocorrelation (18).

Although the “distancing” produced by the mandated business closures and by the restrictions on nonessential out-of-home activities slowed the spread of the COVID-19 virus, the analysis also suggests that business closures played a disproportionately larger role in reducing the positivity rate. This finding can inform the debate over the tradeoffs faced in the development of anticontagion policies and may affect calculations of the net economic cost of those policies (19). The economic disruptions resulting from the mandated business closures, which included a historic increase in the number of people out of work, may be very damaging (20,21). At the same time, those closures led to a considerable decrease in the positivity rate, resulting in fewer serious illnesses (and fatalities) and potentially large reductions in health care costs.

This study has several limitations. The data on testing outcomes at the zip code level did not become available until April 1, 2020 (after the executive order went into effect on March 22). Ideally, the analysis would have used data on positivity rates in the various zip codes both before and after the regulations began to affect behavior. The large change observed in the business activity and stay-at-home indices from the prepandemic baseline might lead to more precise estimates of their impact on the positivity rate.

There are also limitations with the testing data released by the New York City DOH: the testing results may not have been released on a particular day; the counts were sometimes inconsistent across adjacent days; and the date the testing data were reported may differ from the date the test was actually administered. This measurement error likely biases the regression coefficients. If the errors were random, the measured impacts of the business closures and restrictions on out-of-home activities are probably underestimated.

The business activity and stay-at-home indices used in the analysis may have limited informational content. The number of visitors to various points of interest does not directly measure how people who reside in the neighborhood are exposed to and interact with the visitors. The exposure might vary depending on the nature of the point of interest (eg, a park is different than the small corner grocery store). Similarly, the number of smartphone devices that have not left their home location on any given day is an incomplete measure of what social distancing and “shelter-at-home” entails. Moreover, the nature of the New York State on PAUSE executive order produced an interaction between the 2 indices: business closures likely increased the value of the stay-at-home index. This interdependence makes it difficult to forecast the impact of a narrower policy.

The cell phone location data that can potentially increase our understanding of mobility patterns in the population are also imperfectly measured. The people who own the sampled devices (and are captured by the SafeGraph algorithm) may not form a representative sample of the population; the available location data do not adjust for ownership of multiple (or zero) devices; and the definition of the “home location” for any particular device is sensitive to idiosyncratic variation across individuals, such as working a night shift.

Finally, part of the positivity rate variation across zip codes likely arises because COVID-19 testing resources were not allocated randomly across neighborhoods (at least in the initial stage of the pandemic). Although the regression analysis partially addresses this problem by including a vector of zip code fixed effects, these fixed effects only net out the impact of geographic factors that had a constant impact on the positivity rate of the neighborhood at all times. It is possible, however, that the nonrandomness in the allocation of testing resources was addressed as the volume of testing increased in April and May, so that the actual impact of the geographic characteristics presumably captured by the zip code fixed effects would have changed over time.

Further analysis of testing data that might eventually become available at the individual level would help resolve some of these problems. The individual-level data would allow a research design that links test outcomes to both individual and area characteristics.
